# Mn-Doped Spinel for Removing Cr(VI) from Aqueous Solutions: Adsorption Characteristics and Mechanisms

**DOI:** 10.3390/ma16041553

**Published:** 2023-02-13

**Authors:** Manman Lu, Zijian Su, Yuanbo Zhang, Hanquan Zhang, Jia Wang, Qian Li, Tao Jiang

**Affiliations:** 1 School of Resources and Safety Engineering, Wuhan Institute of Technology, Wuhan 430205, China; 2 School of Minerals Processing and Bioengineering, Central South University, Changsha 410083, China

**Keywords:** chromium, reduction, adsorption, MnFe_2_O_4_, DFT calculation, spinel

## Abstract

In this study, the manganese (Mn) was doped in the MnFe_2_O_4_ crystal by the solid-phase synthesis method. Under the optimum conditions (pH = 3), the max removal rate and adsorption quantity of Cr(VI) on MnFe_2_O_4_ adsorbent obtain under pH = 3 were 92.54% and 5.813 mg/g, respectively. The DFT calculation results indicated that the adsorption energy (E_ads_) between HCrO_4_^−^ and MnFe_2_O_4_ is −215.2 KJ/mol. The Cr(VI) is mainly adsorbed on the Mn atoms via chemical bonds in the form of HCrO_4_^−^. The adsorption of Mn on the MnFe_2_O_4_ surface belonged to chemisorption and conformed to the Pseudo-second-order equation. The mechanism investigation indicated that the Mn in MnFe_2_O_4_ has an excellent enhancement effect on the Cr(VI) removal process. The roles of Mn in the Cr(VI) removal process included two parts, providing adsorbing sites and being reductant. Firstly, the Cr(VI) is adsorbed onto the MnFe_2_O_4_ via chemisorption. The Mn in MnFe_2_O_4_ can form ionic bonds with the O atoms of HCrO_4_^−^/CrO_4_^2−^, thus providing the firm adsorbing sites for the Cr(VI). Subsequently, the dissolved Mn(II) can reduce Cr(VI) to Cr(III). The disproportionation of oxidized Mn(III) produced Mn(II), causing Mn(II) to continue to participate in the Cr(VI) reduction. Finally, the reduced Cr(III) is deposited on the MnFe_2_O_4_ surface in the form of Cr(OH)_3_ colloids, which can be separated by magnetic separation.

## 1. Introduction

Chromium (Cr) widely exists in the surface and underground water as a common poisonous heavy metal. The Cr in water mainly comes from industrial discharges, such as leather tanning, electroplating, metal plating, and chemical manufacturing [1,2,3]. There are two primary species of Cr, trivalent (Cr (III)) and hexavalent (Cr (VI)) ions in the aqueous system [4], and the toxicity and mobility of Cr are highly dependent on its existing species [5]. It is widely believed that the toxicity of Cr (VI) is much higher than that of Cr (III), and Cr(VI) is highly carcinogenic and teratogenic [6]. The hypertoxic Cr(VI) in the natural environment could cause a range of diseases and pose a major threat to humans and ecology [7,8]. Therefore, many present researchers have focused on the field of Cr(VI) reduction [9,10]. Among this research, the adsorption-reduction method for Cr(VI) removal has received the most extensive research [11]. Furthermore, some biosorbents have been developed for heavy-metal removal [12].

The current Cr(VI) reduction methods primarily include chemical, electrochemical, photocatalytic, and biological reduction processes [13,14,15,16]. Most of the literature has shown that adsorption is a prerequisite for Cr(VI) reduction [17]. Many adsorbents have been prepared to achieve the adsorption-reduction reaction of Cr(VI) on their surfaces [18,19,20]. Among these adsorbents, metallic oxide, especially spinel-type materials, has been most extensively investigated due to its good magnetic properties, easy synthesis, low cost, and chemical stability. Many published literature studies have verified that Cr(VI) can be reduced by certain low-valence metal ions such as Fe(II) and Mn(II) in spinel materials [21,22]. Mu et al. [23] verified that Mn(II) could promote the Cr(VI) reduction via oxalic acid. In the Cr(VI) reduction process, Cr(VI) oxidized Mn(II) to Mn(III). Then, the oxidized Mn(III) acted as an electron bridge, transferring the electron from oxalic acid to Cr(VI). Another recent paper adopted a synthetic Mn_3_O_4_@ZnO/Mn_3_O_4_ composite to reduce Cr(VI) [24]. As an electron carrier, Mn acted in the role of a catalyst during the Cr(VI) reduction. Another research study elaborated on the adsorption mechanisms of Cr(VI) on the surface of manganese oxide in detail [25]. The researchers found that Cr(VI) adsorption belongs to more than one type of reaction. The Cr(VI) can bind to functional groups by the inner-and outer-sphere chromate complexes under different pH values.

Based on the previous research on the effects of Mn on the Cr(VI) reduction, we synthesized a novel Mn-doped spinel to remove the Cr(VI) from the Cr-bearing solution. The appropriate amount of natural ferromanganese and hematite ore powder was mixed and subsequently roasted under suitable conditions, and Mn replaced the Fe atom at the A site in the Fe_3_O_4_ spinel, forming MnFe_2_O_4_ [26]. Some reports have manifested that the MnFe_2_O_4_ has good adsorbing ability on Cr(VI) [27,28,29,30,31,32]. However, these researchers primarily focused on the adsorption characteristics of Cr(VI) on MnFe_2_O_4_, and the mechanism of Cr(VI) removal had been neglected. In this research, DFT calculation was introduced to reveal the adsorption mechanism of Cr(VI)on the MnFe_2_O_4_ surface. A molecular model was built to study the interaction type between Cr(VI) and the MnFe_2_O_4_ surface. What is more, the MnFe_2_O_4_ material used in the previous research is synthesized from pure chemical raw materials, and its crystallinity, morphology, and impurity content are different from the MnFe_2_O_4_ prepared from natural minerals. Indeed, the MnFe_2_O_4_ is more special as an adsorbent compared with other kinds of spinel because of the non-negligible role of Mn in the Cr(VI) removal process. Some investigations have already manifested that Mn ions in MnFe_2_O_4_ have higher reaction activity compared to the Fe ions in Fe_3_O_4_ [33,34].

The critical point of this study is to deeply analyze the roles of Mn in the Cr(VI) removal process on the MnFe_2_O_4_ surface by means of the XPS test and DFT calculation. Furthermore, the adsorption characteristics of Cr(VI) adsorption on the MnFe_2_O_4_ adsorbent were investigated by the batch adsorption experiments. This study attempts to explain the adsorption mechanisms of the Cr(VI) adsorption on the MnFe_2_O_4_ adsorbent.

## 2. Materials and Methods

### 2.1. Materials

In the previous work, our group invented a novel high-temperature solid-phase synthesis method to prepare the MnFe_2_O_4_ adsorbent [26]. To begin with, the ferromanganese ore and hematite ore powders were evenly mixed, and the stoichiometric ratio of Mn and Fe in mixed raw materials was 1:2. The roasting temperature and time were controlled at 1200 °C and 1 h. The mixture briquettes were broken and ground to −0.074 mm, accounting for 70 wt% after roasting. Then the MnFe_2_O_4_ particles and the gangue minerals were separated by magnetic separation. The obtained MnFe_2_O_4_ particles continued to be nano-ground to the size of 200 nm (d0.5 = 200 nm). The nano-MnFe_2_O_4_ particles were used as a magnetic adsorbent for the Cr(VI) removal.

All chemicals used in this research were of analytical grade. The simulated Cr(VI)-bearing solution was prepared using K_2_Cr_2_O_7_ (Sinopharm Group Co., Ltd., Beijing, China). The pH was adjusted with 0.1 M HCl and 0.1 M NaOH solutions.

### 2.2. Adsorption Characteristics

Adsorption experiments were conducted under isothermal conditions using the batch method. The isothermal adsorption experiments were conducted to investigate the Cr(VI) adsorption characteristics of the MnFe_2_O_4_ adsorbent. A certain weight of the MnFe_2_O_4_ adsorbent was added to an Erlenmeyer flask with a 50 mL Cr(VI) solution to conduct the adsorption experiments. The mixed solutions were oscillated for 24 h to ensure the Cr(VI) achieved equilibrium. Then the filtrates were obtained by filtering through a 0.45 μm membrane filter. The equilibrium concentration of Cr(VI) was measured by ICP-MS analysis. The adsorbing amount of Cr(VI) on the MnFe_2_O_4_ adsorbent under various experimental conditions was calculated according to the following equation:

(1) $$q_{e}=\frac{\left(C_{o}-C_{e}\right)\cdot V}{m}$$

where *q_e_* is the adsorbing amount of Cr(VI) on the MnFe_2_O_4_ adsorbent (mg·g^−1^); *C_o_* and *C_e_* are the initial and equilibrium concentrations of the Cr(VI) solution (mg·L^−1^), respectively; *V* is the volume of the Cr(VI)-bearing solution; and m is the mass of the MnFe_2_O_4_ adsorbent.

The pH effect tests were conducted within the pH range of 1–10. The pH of the solution was adjusted by 0.1 M HCl and 0.1 M NaOH. The initial Cr(VI) concentration and MnFe_2_O_4_ dosage were controlled at 10 mg/L and 3 g/L, respectively. The adsorption kinetics of Cr(VI) on the MnFe_2_O_4_ were investigated by changing the oscillation time of the Cr(VI) adsorption. The oscillation times were changed from 5 to 720 min in this research, and the initial Cr(VI) concentration was controlled at 10 mg/L. The Cr(VI) adsorption data at the various oscillation time were analyzed according to the following two models, the pseudo-first-order equation and the pseudo-second-order equation. The linear forms of these two models are listed below [30]:

(2) $$\ln\left(q_{e}-q_{t}\right)=\ln q_{e}-k_{1}t$$

(3) $$\frac{t}{q_{t}}=\frac{1}{k_{2}q_{e}{}^{2}}+\left(\frac{1}{q_{e}}\right)t$$

where *q_e_* and *q_t_* are the adsorption amounts of Cr(VI) at equilibrium and a certain time t (mg·g^−1^), respectively; *k_1_* and *k_2_* are the rate constants of the pseudo-first-order equation and the pseudo-second-order equation, respectively.

The isothermal adsorption experiments were conducted with the initial Cr(VI) concentration range of 1–100 mg/L. The obtained equilibrium concentrations were fitted via the Langmuir and Freundlich models. The linearized Langmuir and Freundlich isotherm models are expressed as the following equations [31]:

(4) $$C_{e}/q_{e}=C_{e}/q_{m}+1/\left(k_{L}q_{m}\right)$$

(5) $$logq_{e}=logk_{F}+\left(1/n\right)logC_{e}$$

where *q_e_* (mg·g^−1^) is the amount of Cr(VI) adsorbed per weight unit of the MnFe_2_O_4_ adsorbent after equilibrium; *C_e_* (mg·L^−1^) is the equilibrium concentration of Cr(VI) in the solution; *q_m_* (mg·g^−1^) is the maximum adsorption capacity of the adsorption; *K_L_* is a constant related to the heat of adsorption (L·mg^−1^); K_F_ (mg^1−n^·L^n^·g^−1^) is a constant related to the extent of adsorption; and 1/n is related to the intensity of adsorption.

### 2.3. Characterization

The X-ray powder diffraction (XRD) patterns of the synthetic MnFe_2_O_4_ before and after nano-scale grinding were detected by a diffractometer with Cu Kα radiation (RIGAKU D/Max 2500, Tokyo, Japan). The tube current and voltage were 250 mA and 40 kV, the scanning range was 10–70°, and the step size was 0.02°. A vibrating sample magnetometer was adopted to measure the magnetism properties of the MnFe_2_O_4_ adsorbent (PPMS Dynacool, Quantum Design, San Diego, CA, USA). The element composition and binding energies of atomic orbital on the MnFe_2_O_4_ surface before and after adsorbing Cr(VI) were investigated by the XPS test (XPS, Mono 650 μm, 200 W, Al Kα, pass energy 20 eV). The scanning step size was 1.0 eV for the full scan and 0.1 eV for the narrow scan. Zeta potential measurements were conducted using a zetasizer under a wide pH range from 3 to 10 at 25 °C, and the pH value was adjusted using 0.1 M HCl and NaOH. The particle size distribution was characterized by a laser particle analyzer (Malvern Mastersizer 2000, Malvern, UK). The specific surface area and pore volume of the MnFe_2_O_4_ adsorbent were measured by nitrogen adsorption-desorption on a specific surface area with a pore analyzer.

The slab supercell that consists of a (2 × 2) unit cell of MnFe_2_O_4_ (100) was constructed as the substrate. A vacuum of 20 Å was added to eliminate the interaction between two periodic slabs and H atoms were attached to O on the surface in acidic conditions (MnFe_2_O_4_-H (100)). The calculations were conducted under density functional theory, and the Vienna ab initio simulation (VASP) code 1, 2 was used to implement. The initial adsorption site was predicted by the simulated annealing method for locating a good approximation to the global minimum. Projector Augmented Wave (PAW) pseudopotentials are used with a cutoff energy of 400 eV for plane wave expansions. The GGA-PBE5 was used as an exchange-correlation function. The atomic relaxation was performed with a k-mesh of 1 × 1 × 1 until the force on each atom is less than 0.02 eV/Å. Moreover, van der Waals forces between the adsorbate and substrate were also corrected by the DFT-D26 dispersion corrections. After relaxation, a gamma-center k-mesh 2 × 2 × 1 was adopted for the ground state total energy calculation. The adsorption energy (Eads) was evaluated by:

Eads = E(total) − E(sub) − E(ads)(6)

where E(total), E(sub), and E(ads) are the total energy of the MnFe_2_O_4_-H (100)-adsorbate complex, MnFe_2_O_4_-H (100), and adsorbates (HCrO_4_^−^, CrO_4_^2−^ and Cr_2_O_7_^2−^) in the same cell, respectively. According to this definition, a negative value is energetically favorable.

The transfer charge (Δchg) is the net charge on adsorbates:

(7) $$\Delta \mathrm{chg}=\overset{n}{\underset{i=1}{\sum }}A_{i}-A_{i}^{0}$$

where *A_i_* and *A_i_^0^* are the self-consistent charge and the atomic charge of atom *i*, respectively. *n* is the total number of studied adsorbates. The negative value represents electron dissipation to the substrate and positive Δchg represents electron accumulation on the adsorbate.

## 3. Results

### 3.1. Characterization of MnFe_2_O_4_ Adsorbent

**Figure 1:**
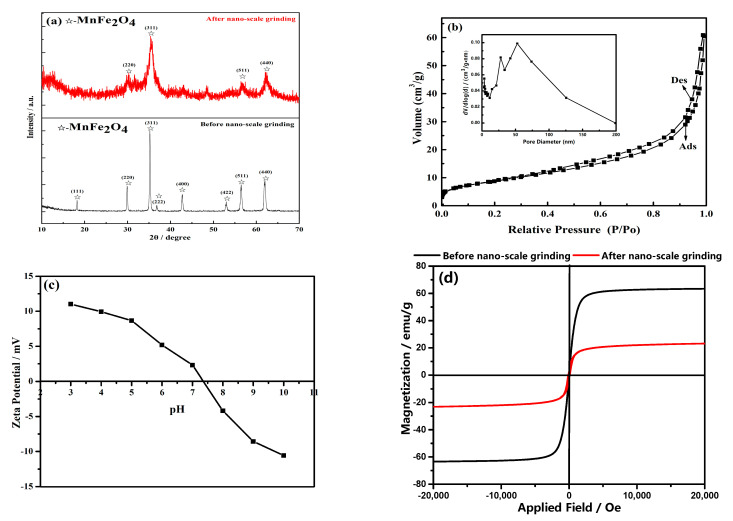
The XRD pattern of MnFe_2_O_4_ before and after nano-scale grinding (**a**); the BET adsorption−desorption curve of MnFe_2_O_4_ adsorbent (**b**); zeta potential of MnFe_2_O_4_ adsorbent (**c**); the magnetization hysteresis loops of MnFe_2_O_4_ before and after nano−scale grinding (**d**).

The effects of nano-scale grinding on the crystallinity and crystal integrity of MnFe_2_O_4_ were investigated by X-ray diffraction patterns. Figure 1a shows that the diffraction peaks at 29.95°, 35.33°, 56.42°, and 61.94° were considered to belong to the 220, 311, 511, and 440 crystal faces of MnFe_2_O_4_ [35], respectively. After grinding, the crystallinity of MnFe_2_O_4_ worsened and certain diffraction peaks disappeared. This result indicated that the strong mechanical grinding would damage the MnFe_2_O_4_ crystal to some extent. The magnetization shown in Figure 1d exhibited that the magnetization intensity of MnFe_2_O_4_ decreased sharply from 63.38 to 23.80 emu/g after grinding. The main reason is that the magnetism of MnFe_2_O_4_ is the macroscopic manifestation of the magnetic properties of many tiny magnetic domains inside it, and as the particle size decreases, the number of internal magnetic domains decreases, resulting in a weakening in the magnetism of nano-size MnFe_2_O_4_ particles. Fortunately, the magnetic saturation curves displayed in Figure 1d manifested that the saturation magnetic of nano-MnFe_2_O_4_ still retained 20 emu/g, illustrating that the MnFe_2_O_4_ adsorbent still had good magnetism and could be recovered by magnetic separation. The BET test manifested that the specific surface area of the MnFe_2_O_4_ adsorbent could achieve 31.997 m^2^/g after nano-scale grinding. The zeta potential results exhibited in Figure 1c illustrated that the pHpzc (zero-point charge pH value) of the MnFe_2_O_4_ is 7.5. The MnFe_2_O_4_ adsorbent was positively charged within the pH range of 3–7.5. Therefore, there is an electrostatic attraction between electropositive MnFe_2_O_4_ particles and chromate (HCrO_4_^−^, CrO_4_^2−^, and Cr_2_O_7_^2−^).

### 3.2. Adsorption Characteristics

#### 3.2.1. Effect of the Initial pH

**Figure 2:**
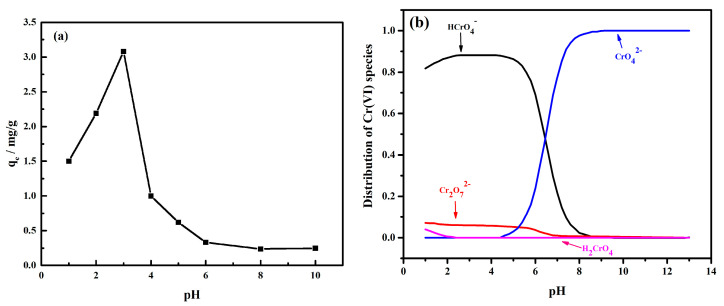
The Cr(VI) removal (**a**) and species (**b**) at various pH values.

In this study, the Cr(VI) adsorption tests were conducted under pH values of 1 to 10. As shown in Figure 2a, the Cr(VI) removal first increased within the pH range of 1–3 and then continued to decline with an increasing pH. The Cr(VI) reduction in the acidic system conformed to the following equations [36,37]:

HCrO_4_^−^ + 3e^−^ + 7H^+^ → Cr^3+^ + 4H_2_O (8)

Cr_2_O_7_^2−^+ 6e^−^ + 14H^+^ → 2Cr^3+^ + 7H_2_O (9)

The hydrogen ions participated in the Cr(VI) reduction, and this indicated that the acidic system is favorable for Cr(VI) reduction. Furthermore, according to the zeta potential tests of MnFe_2_O_4_, the electrostatic attraction between the MnFe_2_O_4_ and chromate is more reliable in a low-pH environment. However, when in a very strong acidic system (pH = 1 and 2), the reduction product Cr(III) could not be deposited on the MnFe_2_O_4_ surface by forming the Cr(OH)_x_ compound due to the neutralization. This is the main reason that the Cr(VI) removal under pH = 1 and 2 is lower than that under pH = 3.

#### 3.2.2. Adsorption Kinetics

**Figure 3:**
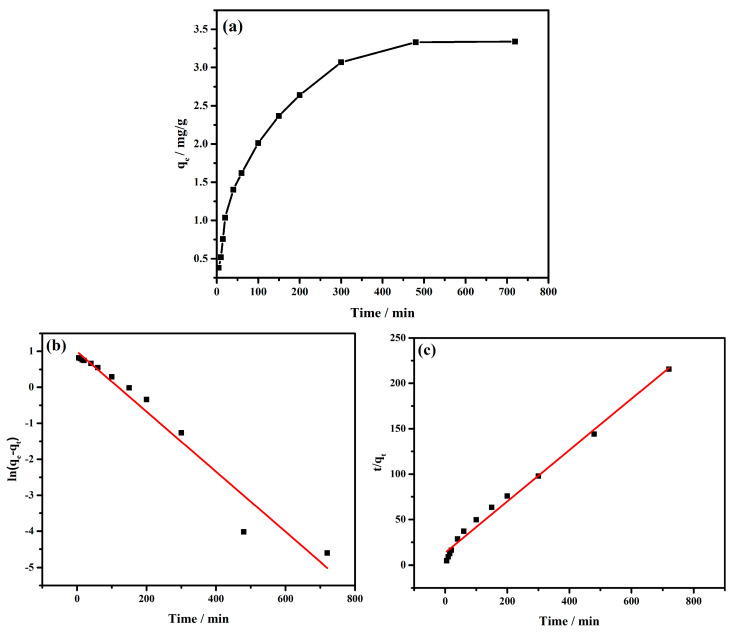
Adsorption kinetics analysis of Cr(VI) on MnFe_2_O_4_ adsorbent: (**a**) Adsorption amount of Cr(VI) at various times; (**b**) fitting line of the pseudo−first−order equation; (**c**) fitting line of the pseudo-second-order equation.

The time needed to reach the adsorption equilibrium was a significant value that decided the Cr(VI) removal efficiency. In this experiment, the Cr(VI) adsorption kinetics was determined by measuring the adsorption amount of Cr(VI) at various times. The adsorption kinetic curve of Cr(VI) is displayed in Figure 3a, and the fitting curves of the pseudo-first-order and pseudo-second-order equations are displayed in Figure 3b,c, respectively. The fitting parameters of the pseudo-first-order equation and pseudo-second-order models are given in Table 1.

The adsorption kinetic curve of Cr(VI) is displayed in Figure 3a, and the fitting curves of the pseudo-first-order and pseudo-second-order equations are displayed in Figure 3b,c, respectively. The fitting parameters of the pseudo-first-order equation and pseudo-second-order models are given in Table 1. The correlation coefficient (R^2^) of these two models indicated that the pseudo-second-order model was more suitable to describe the Cr(VI) adsorption kinetics on MnFe_2_O_4_ adsorbent. The result showed that the Cr(VI) adsorption achieved equilibrium in 480 min, and further extending the adsorption time could hardly affect the adsorption amount of Cr(VI). The above results manifested that Cr(VI) adsorption on the MnFe_2_O_4_ adsorbent belonged to chemisorption [38].

#### 3.2.3. Adsorption Isotherm

**Figure 4:**
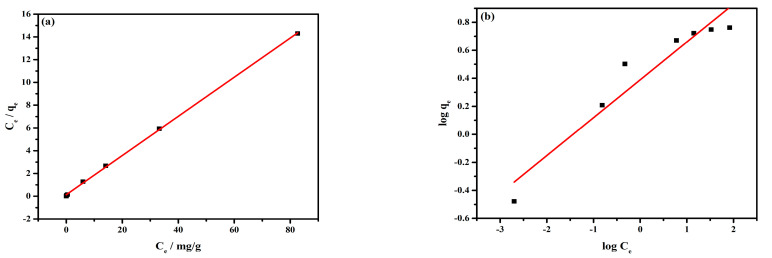
Langmuir (**a**) and Freundlich (**b**) isotherms for the adsorption of Cr(VI) onto the MnFe_2_O_4_ adsorbent.

As many papers reported, the adsorption isotherm model fitting was a common tool to understand the adsorption type between the adsorbent and adsorbate [39]. The Langmuir and Freundlich isotherm models fitting lines and parameters of Cr(VI) adsorption on the MnFe_2_O_4_ surface are shown in Figure 4 and Table 2, respectively.

The results illustrated that the max adsorption quantity of Cr(VI) is 5.813 mg/g. The correlation coefficient (R^2^) of the Langmuir model is higher than that of the Freundlich model, showing that the Cr(VI) adsorption on the MnFe_2_O_4_ surface conformed to the Langmuir model. It was likely that the Cr(VI) adsorption tended to be monolayer adsorption [40]. Compared with other adsorbents, the adsorption capacity of the adsorbent in this study is lower. The main reason is that there are some impurities when using natural minerals for synthesis. What is more, the solid-phase synthetic MnFe_2_O_4_ particles are coarse, while after grinding, their specific surface area is still much smaller than that of the liquid-phase synthetic MnFe_2_O_4_ particles.

### 3.3. Enhancement Mechanism of Mn on Cr(VI) Adsorption and Reduction

#### 3.3.1. Cr(VI) Adsorption Analyses

The Cr(VI) removal process on the MnFe_2_O_4_ surface primarily included the adsorption and reduction process. The Cr(VI) could only be reduced after being adsorbed on the MnFe_2_O_4_ surface. As much of the literature has reported, the DFT calculation is a powerful tool to investigate the interaction reaction between two kinds of molecules [41,42]. In this study, the MnFe_2_O_4_ (100) surface, a typical low-index surface, was used to investigate the role of Mn in the Cr(VI) adsorption process.

**Figure 5:**
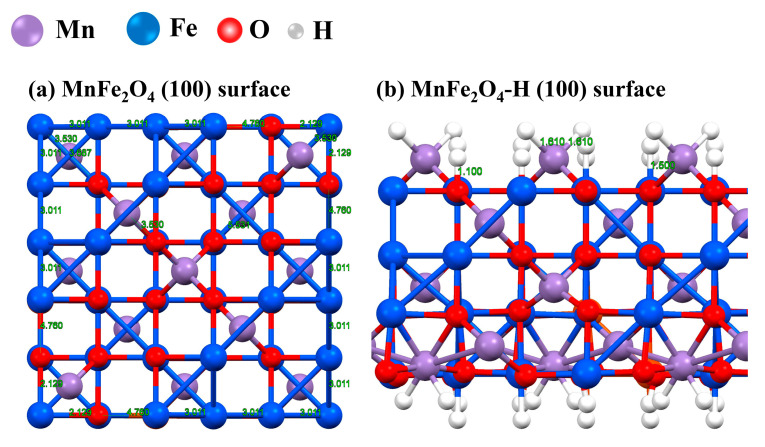
The optimum structure of (**a**) MnFe_2_O_4_ (100) surface and (**b**) MnFe_2_O_4_−H (100) surface.

Figure 5a shows the optimum structure of spinel-type MnFe_2_O_4_, the bond distances of Fe-Fe, Fe-Mn, Fe-O, and Mn-O are 3.011 Å, 3.530 Å, 4.760 Å, and 3.687 Å, respectively. However, according to the stern double-layer theory [43], when MnFe_2_O_4_ is placed in an aqueous solution, a layer of hydrogen or hydroxyl groups adsorbed on the surface of MnFe_2_O_4_ molecules exists, which is called the “positioning ion” [44]. In this study, the Cr(VI) adsorption process primarily occurred in the acidic solution, and the MnFe_2_O_4_-H molecule was dominant compared with MnFe_2_O_4_-OH. We built up a new structure of MnFe_2_O_4_-H by causing the hydrogen ion to adsorb on the surface of the MnFe_2_O_4_ molecule. The optimum structure of MnFe_2_O_4_-H is displayed in Figure 5b. The bond distances of Fe-H, Mn-H, and O-H were 1.500 Å, 1.610 Å, and 1.100 Å, respectively.

**Figure 6:**
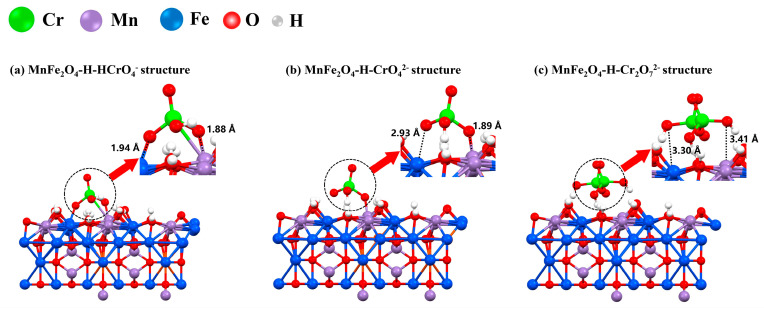
The optimum structure of MnFe_2_O_4_−H−HcrO_4_^−^ (**a**), MnFe_2_O_4_−H−CrO_4_^2−^, (**b**) and MnFe_2_O_4_−H−Cr_2_O_7_^2−^ (**c**).

Next, the groups of HcrO_4_^−^, CrO_4_^2−^, and Cr_2_O_7_^2−^ were selected to conduct DFT calculations. The optimum structures of MnFe_2_O_4_−H−HcrO_4_^−^, MnFe_2_O_4_−H−CrO_4_^2−^, and MnFe_2_O_4_−H−Cr_2_O_7_^2−^ are shown in Figure 6. Moreover, the adsorption energies and the bond distances of HcrO_4_^−^, CrO_4_^2−^, and Cr_2_O_7_^2−^ on MnFe_2_O_4_-H (100) are listed in Table 3.

As shown in Table 4, the adsorption energies of HcrO_4_^−^, CrO_4_^2−^, and Cr_2_O_7_^2−^ on the MnFe_2_O_4_-H surface were −215.2, −200.7, and −116.7 kJ/mol, respectively. The adsorption energy is an indicator of system stability calculated by subtracting the system’s energy before and after adsorption [45]. In general, the higher the adsorption energy is, the more stable the system after adsorption is [46]. Therefore, it can be easily observed that HcrO_4_^−^ was the most easily adsorbed on the MnFe_2_O_4_ adsorbent. Moreover, it can also be observed that from the bond distances displayed in Table 3, in the MnFe_2_O_4_−H−HcrO_4_^−^ system, the O atom of HcrO_4_^−^ formed a chemical bond with the Mn atoms of MnFe_2_O_4_, providing high adsorption energy. As we know, the adsorption process is an essential link to the subsequent reduction [47,48]. The DFT calculation indicated that the HcrO_4_^−^ could be efficiently adsorbed by the Mn atoms of the MnFe_2_O_4_ via the chemical bonds. This efficient adsorption of Cr(VI) created a favorable environment for the subsequent Cr(VI) reduction by the Mn(II).

Subsequently, the charge density distribution was calculated by VASP software to clarify the chemical bond type between the chromate and the MnFe_2_O_4_-H.

**Figure 7:**
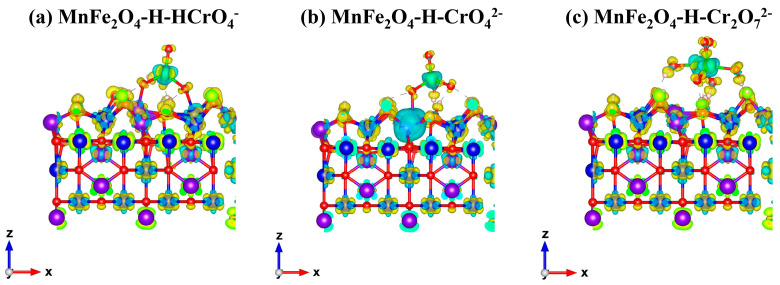
The charge density of MnFe_2_O_4_−H−HcrO_4_^−^ (**a**), MnFe_2_O_4_−H−CrO_4_^2−^, (**b**) and MnFe_2_O_4_−H−Cr_2_O_7_^2−^ (**c**).

In general, the charges would be distributed at both ends of the bond in the electrostatic interaction or ionic bond [49]. In contrast, for the covalent bond, the charge would be distributed in the middle of the bond [50]. The charge density distribution shown in Figure 7 indicates that the charges of the Mn/Fe-O bonds in the MnFe_2_O_4_−H− HcrO_4_^−^ system were distributed at the ends of the bonds. This implied that the Mn/Fe-O bond belonged to the ionic bond. Similarly, CrO_4_^2−^ was also adsorbed on the Mn atom of the MnFe_2_O_4_ adsorbent via the ionic bond, while during the Cr_2_O_7_^2−^ adsorption process, the bonding had little effect on the charge distribution of the two molecules. Therefore, it could be suggested that the adsorption of Cr_2_O_7_^2−^ on the MnFe_2_O_4_ surface is a type of physical adsorption via electrostatic attraction.

#### 3.3.2. Cr(VI) Reduction Analyses

**Figure 8:**
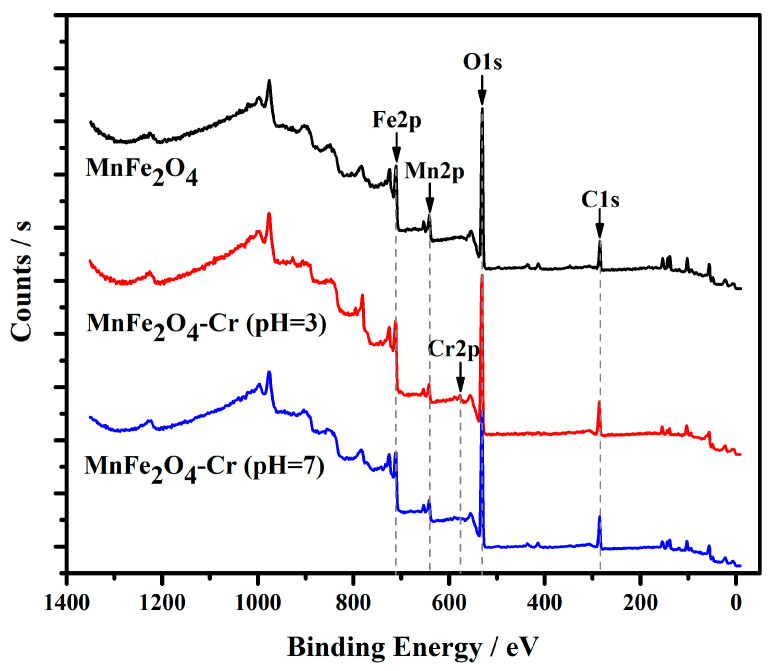
The XPS spectra of MnFe_2_O_4_ adsorbent, MnFe_2_O_4_−Cr (pH = 3), MnFe_2_O_4_-Cr (pH = 7).

The chemical states on the MnFe_2_O_4_ surface before and after the Cr(VI) adsorption were investigated by the XPS test to explain the reduction mechanisms of Cr(VI). The XPS full-scanning spectra of the original MnFe_2_O_4_ adsorbent and MnFe_2_O_4_ adsorbed Cr(VI) under pH = 3 and 7 are shown in Figure 8. The main atom contents on the MnFe_2_O_4_ adsorbent surface before and after adsorbing Cr(VI) are displayed in Table 5.

As shown in Figure 8, there were four primary XPS peaks, 284.8, 532.8, 638.7, and 710.9 eV, belonging to the C1s, O1s, Mn2p, and Fe2p atomic orbitals, respectively. After the Cr(VI) adsorption, the Cr2p peak at 573.6 eV emerged. The results of atom contents showed that the Cr atom content of MnFe_2_O_4_-Cr at pH = 3 was significantly higher than that of MnFe_2_O_4_-Cr at pH = 7 due to the high adsorption quantity of the Cr(VI) under an acidic environment. It is worth noting that the Mn2p atom content declined after Cr(VI) adsorption, implying that the Mn atom in MnFe_2_O_4_ particles was involved in the Cr(VI) reduction reaction. It was inferred that the divalent Mn was oxidized to trivalent Mn by Cr(VI) and entered the solution, resulting in a decrease in the atom content of Mn on the surface of the MnFe_2_O_4_ adsorbent.

**Figure 9:**
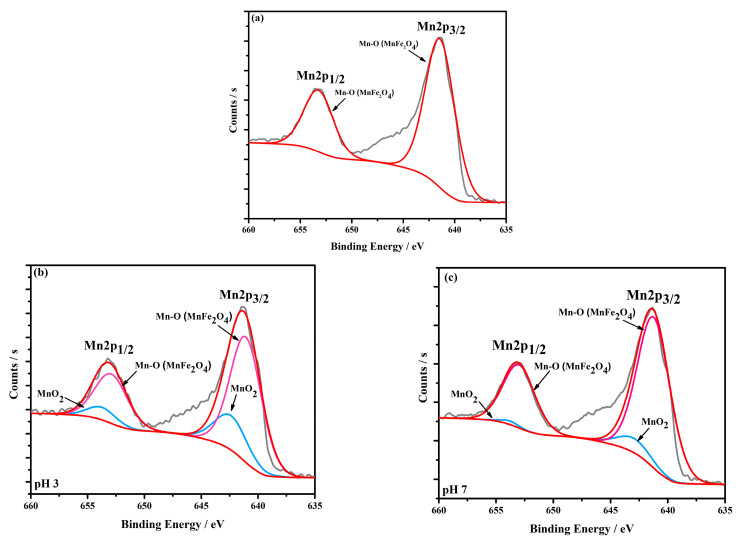
The Mn2p decomposition spectra of MnFe_2_O_4_ (**a**), MnFe_2_O_4_−Cr (pH = 3) (**b**) and MnFe_2_O_4_−Cr (pH = 7) (**c**).

To further investigate the adsorption mechanisms of Cr(VI), the high-resolution spectra of Mn2p and Cr2p were conducted. From the Mn2p spectrum of the original MnFe_2_O_4_ adsorbent shown in Figure 9a, there is only one main peak at 641.5 eV of Mn-O considered to belong to the MnFe_2_O_4_. However, after the Cr(VI) adsorption, the peak of MnO_2_ emerged, indicating that the Mn(II) on the MnFe_2_O_4_ surface might be oxidized to tetravalence Mn by Cr(VI). In fact, Mn(II) was oxidized to Mn(III) first by Cr(VI). However, the Mn(III) was extremely unstable in an acidic solution and was prone to the disproportionation reaction to form Mn(II) and MnO_2_. The resulting Mn(II) could continue to participate in the Cr(VI) reduction, and MnO_2_ was deposited on the MnFe_2_O_4_ surface. The XPS results proved that Mn(II) acted as a reductant in the Cr(VI) reduction process.

**Figure 10:**
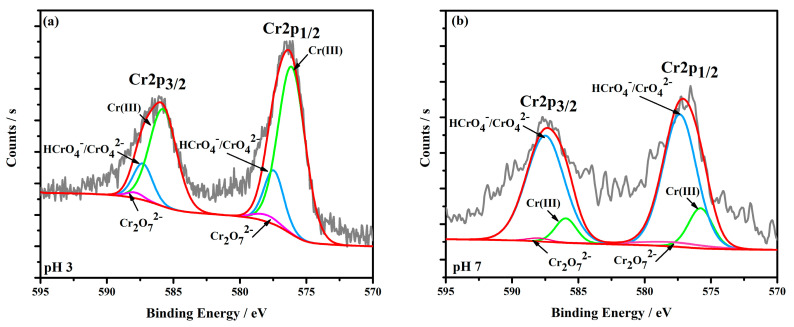
The Cr2p decomposition spectra of MnFe_2_O_4_−Cr at pH = 3 (**a**) and pH = 7 (**b**).

The high-resolution Cr2p spectra of MnFe_2_O_4_-Cr depicted in Figure 10 could be resolved into three individual peaks: The groups of HCrO_4_^−^/CrO_4_^2−^ (577.5 and 587.2 eV), Cr^3+^ (576.2 and 585.9 eV), and Cr_2_O_7_^2−^ (578.1 and 587.8 eV) [51]. Under the pH = 3 condition, the XPS spectrum showed that the main peak of Cr2p on the MnFe_2_O_4_-Cr surface is Cr(III). When the pH value increased to 7, the dominant species of Cr is Cr(VI). It could be seen that from the XPS results, the Cr adsorbed on the MnFe_2_O_4_ surface, mainly in the form of Cr(III) under pH = 3. This result confirmed that most of the Cr(VI) was reduced to Cr(III) by Mn(II) and formed the Cr(OH)_3_ compound on the MnFe_2_O_4_ surface. The rest of the Cr adsorbed onto the MnFe_2_O_4_ surface in the form of Cr(VI) via electrostatic adsorption. According to the XPS results, we could speculate that the Cr(VI) was adsorbed on the surface of the MnFe_2_O_4_ adsorbent in the form of chromate. Subsequently, most of the chromate was reduced to Cr(III) by Mn(II).

### 3.4. The Roles of Mn in Cr(VI) Adsorption and Reduction Process

**Figure 11:**
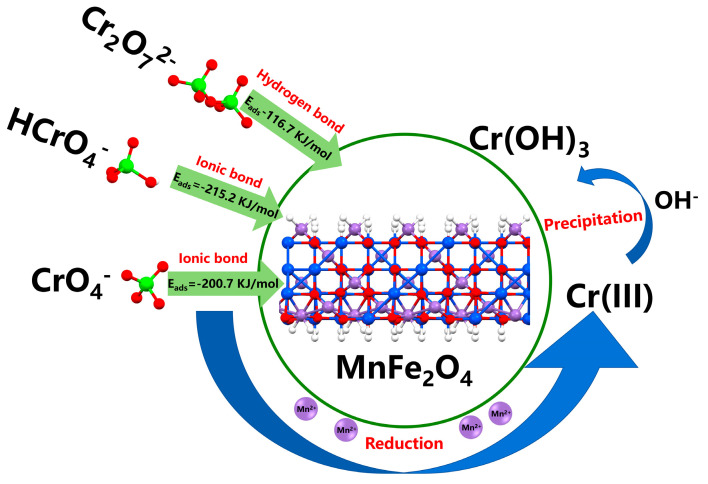
The diagram of the Cr(VI) adsorption and reduction processes.

According to the above results, it could be inferred that the roles of Mn in the Cr(VI) removal process could be divided into two parts, providing the adsorbing sites and being a reductant. The Cr(VI) removal process is explained in Figure 11.

In the Cr(VI) adsorption process, the Mn atoms of the MnFe_2_O_4_ could form a firm ionic bond with the O atoms of HCrO_4_^−^ and CrO_4_^2−^, thus providing the adsorbing sites for the Cr(VI) adsorption. Then, the adsorbed Cr(VI) was reduced by the Mn(II) dissolved on the MnFe_2_O_4_ surface. In the reduction process, the dissolved Mn(II) acted as a reductant. The Mn(II) first oxidized to Mn(III) by Cr(VI). The unstable Mn(III) would cause the disproportionated reaction and formed Mn(II) and MnO_2_ in the aqueous system [52]. The reaction link of the Cr(VI) reduction by Mn(II) can be described by the following equations:

(10) $$3Mn^{2+}+HCrO_{4}^{-}+7H^{+}\;\to \;3Mn^{3+}+Cr^{3+}+4H_{2}O$$

(11) $$Cr^{3+}+3OH^{-}\;\to Cr{\left(OH\right)}_{3}$$

(12) $$2Mn^{3+}+2H_{2}O\;\to Mn^{2+}+MnO_{2}+4H^{+}$$

After Cr(VI) reduction, the obtained Cr(III) was complexed with the hydroxy groups to form the inner-sphere complex [53]. The Cr(OH)_3_ precipitate was deposited on the MnFe_2_O_4_ surface and separated by magnetic separation.

## 4. Conclusions

The Cr(VI) could be removed efficiently by the MnFe_2_O_4_ adsorbent. The adsorption process belonged to chemisorption. The mechanism research showed that the roles of Mn in the Cr(VI) removal process could be divided into two parts, providing adsorbing sites and being a reductant. The adsorption mechanisms of the Cr(VI) on the MnFe_2_O_4_ adsorbent was the formation of ionic bonds between O atoms of HCrO_4_^−^/CrO_4_^2−^ and the Mn/Fe atoms of the MnFe_2_O_4_ adsorbent, which provided firm adsorbing sites for the Cr(VI). Subsequently, the adsorbed Cr(VI) was reduced by Mn(II) dissolved from the MnFe_2_O_4_ surface. The reduced Cr(III) would form the Cr(OH)_3_ colloids deposited on the MnFe_2_O_4_ surface, then be removed by magnetic separation. The present work clarified the roles of Mn in the Cr(VI) removal process and provided the removal mechanisms of Cr(VI) on the MnFe_2_O_4_ adsorbent.

## Figures and Tables

**Table 1:** Kinetic fitting parameters for the pseudo-first-order and pseudo-second-order equations for Cr(VI) adsorption onto the MnFe_2_O_4_ adsorbent.

		Pseudo-First-Order Equation			Pseudo-Second-Order Equation		
Adsorbate	qe (mg/g)	qe,c (mg/g)	k1 (min^−1^)	R^2^	qe,c (mg/g)	K2 (g/mg·min^−1^)	R^2^
Cr(VI)	3335	2698	0.00834	0.959	3538	0.00597	0.989

**Table 2:** The parameters for Langmuir and Freundlich models for Cr(VI) adsorption onto the MnFe_2_O_4_ adsorbent.

T (K)	Langmuir			Freundlich		
	qm (mg/g)	kL (L·mg^−1^)	R^2^	kF (mg^1−n^·Ln·g^−1^)	1/n	R^2^
298	5813	1282	0.9995	2448	0.270	0.9121

**Table 3:** Comparison of the results of this research with other related research.

Adsorbents	Metal Ions	Synthetic Raw Materials	Adsorption Capacity/mg/g	Refs
MnFe_2_O_4_	Cr(VI)	Natural minerals	5813	This study
BCMFC	Cr(VI)	Chemical reagents	178.6	[27]
Nano-MnFe_2_O_4_	Cr(VI)	Chemical reagents	31.5	[25]
MnFe_2_O_4_/FeS_X−0.5_	Cr(VI)	Chemical reagents	43.36	[29]
MFO/C NPs	Cr(VI)	Chemical reagents	73.26	[30]

**Table 4:** The adsorption energy, the shortest distance between Mn/Fe and O, and the charge transfer between MnFe_2_O_4_−H (100) and adsorbates.

	Eads/kJ/mol	D(Mn-O)/Å	D(Fe-O)/Å	Δchg
HcrO_4_^−^	−215.2	1.88	1.94	0.609
CrO_4_^2−^	−200.7	1.89	2.93	0.524
Cr_2_O_7_^2−^	−116.7	3.41	3.30	0.032

**Table 5:** Atom contents of the surfaces of the three samples.

Elements	Atom Content of MnFe_2_O_4_/%	Atom Content of MnFe_2_O_4_-Cr (pH3)/%	Atom Content of MnFe_2_O_4_-Cr (pH7)/%
C1s	24.08	30.02	29.76
O1s	60.65	56.00	55.58
Fe2p	11.73	11.17	11.35
Mn2p	3.55	2.03	3.11
Cr2p	None	0.78	0.19

## Data Availability

The data is unavailable due to privacy.
